# Verschiedene immunologische Typen der CRSwNP im Kontext der neuen Europäischen EAACI-Nomenklatur: Teil 1: Hypersensitivitätsreaktionen vom Typ IVa–c als Korrelat zu T1-, T2- und T3-Endotypen

**DOI:** 10.1007/s00106-025-01600-9

**Published:** 2025-04-08

**Authors:** L. Klimek, S. Becker, B. Haxel, M. Cuevas, P. Huber, A. Chaker, O. Pfaar, M. Laudien, C. Beutner, J. Hagemann, U. Förster-Ruhrmann, H. Olze, B. P. Ernst, A. Beule, C. Rudack, A. S. Hoffmann, C. Betz, M. Gröger

**Affiliations:** 1 Zentrum für Rhinologie und Allergologie, Wiesbaden, Deutschland; 2 Klinik für Hals‑, Nasen- und Ohrenheilkunde, Universitätsmedizin Tübingen, Tübingen, Deutschland; 3 Klinik für Hals‑, Nasen- und Ohrenheilkunde, Universitätsmedizin Mainz, Mainz, Deutschland; 4 Klinik und Poliklinik für HNO-Heilkunde, Universitätsklinikum Carl Gustav Carus, TU Dresden, Dresden, Deutschland; 5 Klinik und Poliklinik für Hals-Nasen- Ohrenheilkunde, Klinikum der Ludwig-Maximilians-Universität, München, Deutschland; 6 TUM School of Medicine and Health, Klinikum rechts der Isar, HNO-Klinik und Zentrum für Allergie und Umwelt, Technische Universität München, München, Deutschland; 7 Klinik für Hals‑, Nasen-und Ohrenheilkunde, Universitätsklinikum Gießen und Marburg GmbH, Standort Marburg, Marburg, Deutschland; 8 Klinik für Hals‑, Nasen- und Ohrenheilkunde, Universitätsklinikum Kiel, Kiel, Deutschland; 9 Klinik für Dermatologie, Venerologie und Allergologie, Universitätsmedizin Göttingen, Göttingen, Deutschland; 10 Klinik für Hals‑, Nasen- und Ohrenheilkunde, Universitätsklinikum Charité, Berlin, Deutschland; 11 Klinik für Hals‑, Nasen- und Ohrenheilkunde, Universitätsmedizin Frankfurt/M, Frankfurt/M, Deutschland; 12 Klinik für Hals‑, Nasen- und Ohrenheilkunde, Universitätsklinikum Münster, Münster, Deutschland; 13 Klinik für Hals‑, Nasen- und Ohrenheilkunde, Universitätsklinikum UKE, Hamburg, Deutschland

**Keywords:** Mukosale Immunantwort, Zytokinprofile, Entzündungsmechanismen, Hyperreaktivität, Personalisierte Therapie, Mucosal immune response, Cytokine profiles, Inflammatory mechanisms, Hyperreactivity, Personalized therapy

## Abstract

**Hintergrund:**

Die chronische Rhinosinusitis (CRS) betrifft bis zu 11 % der Bevölkerung in Europa und den USA und zählt zu den häufigsten chronischen Erkrankungen. Die Klassifizierung nach immunologischen Endotypen, insbesondere des T2-Endotyps, gewinnt zunehmend an Bedeutung. Diese basiert auf der Hypersensitivitätslehre nach Coombs und Gell, die zellvermittelte Typ-IV-Reaktionen als T1-, T2- und T3-Endotypen einteilt. Bei der chronischen Rhinosinusitis mit Nasenpolypen (CRSwNP) spielen genetische und epigenetische Veränderungen des mukosalen Immunsystems eine wesentliche Rolle. Die Identifizierung spezifischer Endotypen ermöglicht es, die Heterogenität der Erkrankung besser zu verstehen und maßgeschneiderte Therapieansätze zu entwickeln. Ziel dieser Arbeit ist es, die zugrunde liegenden immunologischen Mechanismen zu systematisieren und deren Relevanz für Diagnostik und Therapie darzustellen

**Methodik:**

Die Europäische Akademie für Allergie und klinische Immunologie (EAACI) veröffentlichte kürzlich eine aktualisierte Nomenklatur für immunologische Überempfindlichkeitsreaktionen. Die ursprünglich von Coombs und Gell klassifizierten Antikörper-vermittelten Reaktionen (Typ I–III) wurden erweitert. Die zellvermittelten Reaktionen umfassen nun: Typ IVa (T1) → Th1-dominierte Reaktionen; Typ IVb (T2) → Th2-dominierte Reaktionen; Typ IVc (T3) → Th17-dominierte Reaktionen. Diese neuen Erkenntnisse zu T1-, T2- und T3-Signalwegen bilden die Grundlage dieser Arbeit. Weitere Mechanismen wie epitheliale Barrieredefekte (Typ V), chemische Reaktionen (Typ VI) und stoffwechselbedingte Immundysregulationen (Typ VII) werden separat behandelt.

**Ergebnisse:**

Die Endotypisierung zeigt deutliche regionale Unterschiede: Der T2-(Th2-hoch‑) Endotyp, der in Europa, Nord- und Südamerika sowie Australien dominiert, zeichnet sich durch erhöhte Th2-Zytokine (IL‑4, IL‑5, IL-13) und eosinophile Entzündungen aus. Der T1-(Th1-hoch‑) Endotyp zeigt eine dominante Interferon-Gamma-Aktivität und eine nichteosinophile, meist neutrophile Entzündung. Der T3-(Th17-hoch‑) Endotyp ist durch eine erhöhte IL-17-Präsenz gekennzeichnet und tritt sowohl bei eosinophiler als auch nichteosinophiler CRSwNP auf.

**Schlussfolgerungen:**

Bei CRSwNP-Patienten können alle drei Hyperreaktivitäts-Endotypen (T1, T2, T3) isoliert oder kombiniert auftreten. Der T2-Endotyp ist in Europa am häufigsten. Eine gezielte Endotypisierung ermöglicht differenzierte Therapieansätze und neue Behandlungsoptionen.

## Abstract (en)

**Background:**

Chronic rhinosinusitis (CRS) affects up to 11% of the population in Europe and the USA, making it one of the most common chronic diseases. The classification of immunological endotypes, particularly the T2 endotype, is gaining increasing importance. This classification is based on the Coombs and Gell hypersensitivity model, which categorizes cell-mediated type IV reactions into T1, T2, and T3 endotypes. In chronic rhinosinusitis with nasal polyps (CRSwNP), genetic and epigenetic alterations in the mucosal immune system play a key role. Identifying specific endotypes helps to better understand the heterogeneity of the disease and develop tailored treatment approaches. This paper aims to systematize the underlying immunological mechanisms and highlight their relevance for diagnosis and therapy.

**Methods:**

The European Academy of Allergy and Clinical Immunology (EAACI) recently published an updated nomenclature for immunological hypersensitivity reactions. The original Coombs and Gell classification of antibody-mediated reactions (type I–III) has been expanded. Cell-mediated reactions now include: type IVa (T1) → Th1-dominated reactions; type IVb (T2) → Th2-dominated reactions; type IVc (T3) → Th17-dominated reactions. These new insights into T1, T2, and T3 signaling pathways form the basis of this study. Additional mechanisms such as epithelial barrier defects (type V), chemical reactions (type VI), and metabolism-related immune dysregulations (type VII) are addressed separately.

**Results:**

Endotyping reveals distinct regional differences: The T2 (Th2-high) endotype, predominant in Europe, North and South America, and Australia, is characterized by elevated Th2 cytokines (IL‑4, IL‑5, IL-13) and eosinophilic inflammation. The T1 (Th1-high) endotype shows dominant interferon-gamma activity and non-eosinophilic, mainly neutrophilic inflammation. The T3 (Th17-high) endotype is defined by increased IL-17 presence and can occur in both eosinophilic and non-eosinophilic CRSwNP.

**Conclusion:**

In CRSwNP patients, all three hyperreactivity endotypes (T1, T2, T3) can occur individually or in combination. The T2 endotype is the most common in Europe. Targeted endotyping enables differentiated treatment approaches and novel therapeutic options.

Die chronische Rhinosinusitis (CRS) weist in Europa und den USA eine Prävalenz von bis zu 11 % auf und gehört somit zu den häufigsten chronischen Erkrankungen überhaupt [18]. Die Diagnose CRS beruht üblicherweise auf klinischen Parametern. Die klinische Charakterisierung der CRS basiert auf dem Bestehen von mindestens zwei der Hauptsymptome (Gesichts‑/Kopfdruck, nasale Obstruktion, Hyposmie/Anosmie und/oder Nasensekretion), oder einem Hauptsymptom und mindestens 2 Nebensymptomen (Kopfschmerz, Fieber, Halitosis, Husten, Zahnschmerzen, Abgeschlagenheit und Ohrdruck) über einen Zeitraum von mehr als 12 Wochen. HNO-ärztliche Leitlinien fordern zudem einen endoskopischen und/oder radiologischen Nachweis entzündlichen Gewebes zusätzlich zu o. g. Kriterien. Die deutsche S2k-Leitlinie Rhinosinusitis (AWMF 017/049 [HNO] und AWMF 053-012 [DEGAM]) unterscheidet zwischen einer akuten, einer rezidivierenden akuten und einer chronischen Rhinosinusitis (RS).

Demnach ist eine CRS definiert durch ihren zeitlichen Verlauf von typischen Symptomen, die länger als 12 Wochen andauern und dem Vorliegen von typischen Entzündungszeichen, basierend auf endoskopischen Untersuchungen der Nasenhöhle oder bildgebenden Verfahren. Unterschieden wird zwischen einer CRS mit (CRSwNP) und ohne Polypen (CRSsNP). Die CRSwNP ist mit einer Prävalenz von bis zu 4 % deutlich seltener als die CRSsNP, weist jedoch meist einen schwereren Krankheitsverlauf auf [18]. CRSwNP und CRSsNP sind für sich gesehen jeweils keine einheitlichen Krankheitsbilder, da innerhalb dieser Phänotypen verschiedene Pathomechanismen existieren, die zu unterschiedlichen inflammatorischen Reaktionen der Mukosa führen, welche als Endotypen bezeichnet werden [1]. Das Verständnis der hier dargelegten pathophysiologischen Grundlagen soll in der Zukunft eine präzise, individualisierte Therapie ermöglichen.

Bei der CRSwNP spielen Inhalationsallergien eher eine untergeordnete Rolle [2–5], wohingegen bei der CRSsNP Sensibilisierungen vor allem auf Milbenantigene ursächlich diskutiert werden und entsprechende Therapieoptionen bedacht und angewendet werden sollten [6–17]. Vielmehr sind bei der CRSwNP fehlgeleitete immunologische Hyperreaktivitätsreaktionen in der Mukosa der Nasennebenhöhlen entscheidend, die in diesem Beitrag näher beleuchtet werden [18–21].

## Methodik

Der vorliegenden Publikation liegt ein Positionspapier der europäischen Akademie für Allergie und klinische Immunologie (EAACI) zugrunde, in dem eine modernisierte und erweiterte Klassifikation von Hypersensitivitätsreaktionen vorgestellt wird [22]. Diese wurde inzwischen auch an das deutsche Gesundheitssystem angepasst [23–25]. Die Bezeichnung „Überempfindlichkeit“ wurde erstmals 1963 von Robin Coombs und Philip George Houthem Gell eingeführt. Überempfindlichkeit bezieht sich auf eine unerwünschte, unangenehme oder schädliche Reaktion, die aus einer Überreaktion der adaptiven Immunantwort resultiert. Sie umfasst sowohl Allergien, die durch äußere Reize ausgelöst werden, als auch Autoimmunität, die auf intrinsische Reize zurückzuführen ist. Typischerweise setzen Überempfindlichkeitsreaktionen einen vorsensibilisierten (Immun‑)Zustand des Organismus voraus (sekundäre Immunantwort). Nach Coombs und Gell wurden Überempfindlichkeitsreaktionen in vier Typen eingeteilt: Typ I: unmittelbar (IgE-vermittelt), Typ II: zytotoxisch (Antikörper- und Fc-Rezeptor-vermittelt, zellulär), Typ III: Immunkomplex-vermittelt, und Typ IV: verzögert (T-Zell-vermittelt) [22–26]. Diese T‑Zell-vermittelten Typ-IV-Reaktionen und ihre Anwendung auf die Endotyp-Klassifizierung der CRS sind Gegenstand dieser Übersichtsarbeit.

## Ergebnisse

### Historische Entwicklung

Die Bezeichnung „Überempfindlichkeitsreaktionen“ („hypersensitivity reactions“) wurde erstmals 1963 von Coombs und Gell eingeführt. Definiert wird hierdurch eine unerwünschte oder schädliche Reaktion des adaptiven Immunsystems [22–25]. Im Jahr 2001 veröffentlichte die Nomenklatur-Taskforce der EAACI unter der Leitung von S. Gunnar Johansson eine neue Nomenklatur für Allergien [22]. In diesem Dokument wurden Überempfindlichkeiten in die folgenden Kategorien eingeteilt: IgE-vermittelte Reaktionen, zu denen atopische und nichtatopische Erkrankungen (Insektenstiche, Helminthen, Medikamente) gehören; nicht-IgE-vermittelte Störungen, bei denen es sich um zellvermittelte Reaktionen handelt, an denen T‑Lymphozyten (Kontaktdermatitis), IgG-vermittelte (allergische Alveolitis) und andere Immunzellen, z. B. Eosinophile Granulozyten (Gastroenteropathie), beteiligt sind; und nichtallergische Reaktionen, an denen keine Immunmechanismen beteiligt sind [22].

Werner Pichler schlug eine weitere Unterteilung der Überempfindlichkeitsreaktionen vom Typ IV vor, die auf den Schlüsselzellen, Zytokinen und Chemokinen basiert [22]: Typ IVa (Tuberkulinreaktion), bei der Monozyten und Makrophagen (Mφ) bevorzugt aktiviert und rekrutiert werden (mit der Untervariante der granulomatösen Reaktion); Typ IVb, bei welcher Eosinophile und T2-Helferzellen bevorzugt aktiviert und rekrutiert werden; Typ IVc, die durch die zytotoxischen Funktionen von CD8+-T-Zellen vermittelt wird; Typ IVd, bei der Neutrophile bevorzugt aktiviert und rekrutiert werden. Aufgrund des aktuellen Verständnisses, dass CD8^+^-Zellen sehr vielfältig und analog zu CD4^+^-Zelluntergruppen sein können – CD8^+^-Untergruppe 1 (‑Tc1), CD8^+^-Untergruppe 2 (‑Tc2), CD8^+^-Untergruppe 17 (‑Tc17), regulatorische CD8^+^-Untergruppe – (CD8+ Treg) –, wurde dieses Konzept im EAACI-Positionspapier geändert [22]. Somit wurden die ursprünglich von Coombs und Gell klassifizierten Hyperreaktivitäts-Reaktionen erweitert, und zellvermittelte Reaktionen werden als Typ IVa (T1), Typ IVb (T2) und Typ IVc (T3) klassifiziert, deren immunologische Grundlagen und Abläufe nachfolgend dargestellt werden sollen.

### Immunologische Aspekte der CRS

T‑Lymphozyten stehen im Mittelpunkt der Regulation dieser Immunreaktionen, die auch für die Immunpathogenese der CRS eine wesentliche Rolle spielen. CD4^+^-T-Zellen können sich u. a. in T‑Helfer-, z. B. Th1, Th2, Th9, Th17, Th22, und follikuläre T‑Helfer(TFH)-Effektorzellen weiterentwickeln [27, 28]. Persistierende Entzündungsprozesse können die Balance zwischen diesen Th-Subtypen verändern. Bei der CRS können sowohl Cluster mit „reinem“ Entzündungstyp als auch Mischformen gefunden werden [29]. Am häufigsten zeigt sich eine eosinophile, Th2-dominierte Zellinfiltration [18, 21]. Der Entzündungsprozess ist hier durch die Produktion von Interleukin(IL)-4 und IL‑5 durch diese Th2-Zellen sowie durch hohe Spiegel an eosinophilem kationischem Protein (ECP) und Eotaxin-1/-2/-3 gekennzeichnet. IL‑4 wird hauptsächlich von Th2-Zellen produziert und kann die Differenzierung von CD4^+^-T-Zellen in Th2-Zellen fördern und die IFN-γ-Produktion und Th1-Antwort inhibieren. IL‑5 ist das wichtigste Eosinophilen-aktivierende Zytokin und fördert das Überleben von reifen Eosinophilen im Gewebe, ECP und Eotaxin begünstigen die Anlockung und Aktivierung von Eosinophilen.

#### Ablauf von Typ-IV- oder zellvermittelten Reaktionen

Gedächtnis-T-Lymphozyten, die mit ILC, NK-T-Zellen, NK-Zellen, neutrophilen Granulozyten, eosinophilen Granulozyten und M1-Makrophagen interagieren, lösen Typ-IV-Reaktionen aus. Verschiedene Untergruppen von T‑Zellen vermitteln Typ-IV-Reaktionen über unterschiedliche spezifische Wege und weisen ein hohes Maß an Heterogenität auf, was die unterschiedlichen phänotypischen Merkmale von Gedächtnis-Lymphozyten widerspiegelt. Einige Krankheitsmechanismen lassen sich nur durch das Zusammenwirken mehrerer Subtypen der Typ-IV-Hypersensitivität erklären.

### Typ-IVa-Hypersensitivitätsreaktionen

#### T1-Immunantwort und T1-Endotyp

Der T1-Endotyp wird bei der CRS von Th1- und Tc1-Gedächtniszellen vermittelt, die ihren Phänotyp durch die Exposition gegenüber IL-12, IL-23 und IFN‑γ von Antigen-präsentierenden Zellen (APC) erhalten [22]. Th1-Zellen produzieren große Mengen an IFN‑γ, Lymphotoxin und Tumornekrosefaktor-alpha (TNF-α), die viele Krankheitsmechanismen vermitteln, darunter die Granulombildung, die Synthese von IgG 1 und IgG 3 durch B‑Zellen und die Aktivierung der T‑Zell-Zytotoxizität [30]. Die Immunantwort der Gedächtniszellen bei Typ-IVa-Reaktionen wird durch angeborene Immunzellen verstärkt, zu denen unter anderem ILC1 und klassisch aktivierte Mφ gehören [31]. Aktivierte Mφ setzen verschiedene Entzündungsmediatoren wie reaktive Sauerstoffspezies (ROS), Proteasen und proinflammatorische Zytokine frei, die zur Gewebeschädigung am Ort der Antigenexposition beitragen.

Darüber hinaus ändern die Th2-Zellen bei T2-abhängigen Atemwegserkrankungen nach ihrer Wanderung ins Gewebe ihren Phänotyp und produzieren und exprimieren T1-Effektorzytokine: IFN‑γ, TNF‑α sowie Fas-Ligand und andere Todessignale, die eine Apoptose der Epithelzellen mit anschließendem Remodelling auslösen [32, 33].

**Abb. 1:**
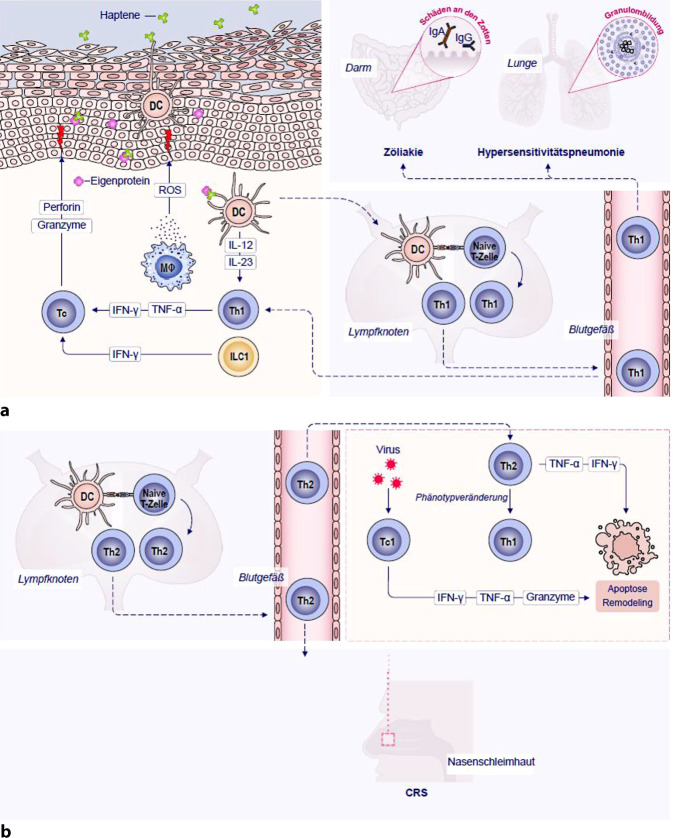
Mechanismen der Typ-IVa-Hypersensitivität (**a**) beim T1-Endotyp der CRS (**b**): APC präsentieren Antigen/Hapten an Th1-Gedächtniszellen, die bei Zytokinexposition ihren Phänotyp annehmen, der zur Aktivierung, Proliferation und Produktion von IFN‑γ und TNF‑α führt. Diese Zytokine rekrutieren und aktivieren Immunzellen, was zu Entzündungen und Gewebeschäden führt. Mφ produzieren ROI; Tc und NK setzen Granzyme und Perforine frei. Angeborene Immunzellen, insbesondere ILC1, verstärken die Reaktion, indem sie eine große Menge IFN‑γ produzieren. Die klinische Manifestation der Typ-IVa-Reaktion ist typisch für ACD, bei der das auslösende Hapten ein kleines Molekül ist, das sich mit einem Wirtsprotein (z. B. einem epidermalen Protein) verbinden muss, um in einem als Harmonisierung bezeichneten Prozess immunogen zu werden. Auf intestinaler Ebene wird die Zöliakie durch Gliadin-spezifische Th1-Zellen vermittelt, die nach dem Verzehr von Weizen und anderen Getreidesorten eine Darmentzündung auslösen. Die chronische Darmentzündung mit Schädigung der Darmzotten geht mit der Bildung von IgA- und IgG-Antikörpern gegen Gewebeproteine einher: Anti-Gewebe-Transglutaminase-AB (tTG-IgA), Anti-Endomysial-AB (EMA-IgA), Anti-deamidierte Gliadin-Peptide-AB (DGP-IgA und DGP-IgG), wodurch die Krankheit zu einer gemischten Allergie-Autoimmunerkrankung wird. Die chronische Phase der HP richtet sich gegen luftgetragene Allergene, die eine Entzündung im Lungenparenchym auslösen, was schließlich zur Bildung von Lungengranulomen und zur Vernarbung (Fibrose) des Lungengewebes führt. **b** Th2-Zellen, die in die Nasenschleimhaut einwandern, produzieren häufig T1-Effektorzytokine: IFN‑γ, TNF‑α und Fas-Ligand (Todessignale), die eine Apoptose des nasalen Schleimhautepithels auslösen können, gefolgt von einer Umstrukturierung. Die CD8^+^-T-Zellen (Tc1), die auf Virusinfektionen reagieren, können zur Gewebeentzündung und -umgestaltung beitragen. Viren aktivieren Tc1-Zellen, die IFN‑γ, Granzyme usw. produzieren und eine Hyperreaktivität der Atemwege auslösen. *ACD* allergische Kontaktdermatitis/allergisches Kontakt-Ekzem; *AD* atopische Dermatitis; *AR* allergische Rhinitis; *CRS* chronische Rhinosinusitis; *DC* dendritische Zelle; *IFN‑γ* Interferon-gamma; *IL* Interleukin; *ILC1* angeborene lymphoide Zelle vom Typ 1; *HP* Hypersensitivitätspneumonitis; *Mφ* Makrophage; *ROI* reaktive Sauerstoffspezies; *Th naive/1/2* T-Helfer-Lymphozyten vom naive/1/2-Typ; *Tc* zytotoxische Lymphozyten; *TNF‑α* Tumornekrosefaktor-alpha

Zytotoxische CD8^+^-Tc1-Gedächtniszellen sind auch an Typ-IVa-Reaktionen beteiligt [34]. Zytotoxische T‑Gedächtniszellen differenzieren sich in der Regel, wenn sie dem von APC und Th1-Zellen freigesetzten IFN‑γ ausgesetzt sind [35]. Tc-Zellen produzieren große Mengen von IFN‑γ und vermitteln viele Entzündungsmechanismen [36]. Die Aktivierung von Tc-Gedächtniszellen unterscheidet sich von derjenigen der Th-Gedächtniszellen. Während Th-Zellen nur am Ort der Entzündung durch APC reaktiviert werden können, die MHC-II-Moleküle exprimieren und exogene antigene Peptide präsentieren, können Tc-Zellen lokal durch jede Zelle reaktiviert werden, die MHC-I-Moleküle exprimiert und endogene antigene Peptide präsentiert, einschließlich Stromazellen [37]. Nach der Aktivierung erhöhen Tc1-Gedächtniszellen die Expression von Perforin und Granzym B und vermitteln so die Lyse der Zelle, die das Antigen im Zusammenhang mit MHC-I-Molekülen exprimiert [35, 38, 39]. Darüber hinaus spielen TNF‑α, Fas-Ligand, Tumornekrosefaktor(TNF)-like Weak Inducer of Apoptosis (TWEAK) und „TNF-related apoptosis-inducing ligand“ (TRAIL) eine Rolle bei Gewebeschäden, insbesondere bei der Apoptose von Epithelzellen [40, 41]. Es hat sich gezeigt, dass CD8^+^-T-Zellen, die eine entscheidende Rolle bei der antiviralen Immunabwehr spielen, auch chronische Entzündungen und Umbauprozesse auslösen können. Rhinoviren, respiratorische Synzytialviren (RSV), Influenzaviren, Parainfluenzaviren, humane Metapneumoviren oder Coronaviren (SARS-CoV-2) aktivieren Tc1-Zellen, die IFN‑γ, Granzyme usw. produzieren, was zu Gewebeschäden führt und auch eine Hyperreaktivität der Atemwege auslösen kann (Abb. 1).

### Typ-IVb-Hypersensitivitätsreaktionen

#### T2-Immunantwort und T2-Endotyp

Der T2-Endotyp ist die charakteristischste Ausprägung der Immunreaktion bei der CRS (T2-Immunantwort). Th2-Zellen, ILC2, NK-T-Zellen, Eosinophile und eine Untergruppe von Mφ sind die Hauptakteure der Typ-IVb-T2-Immunantwort.

**Abb. 2:**
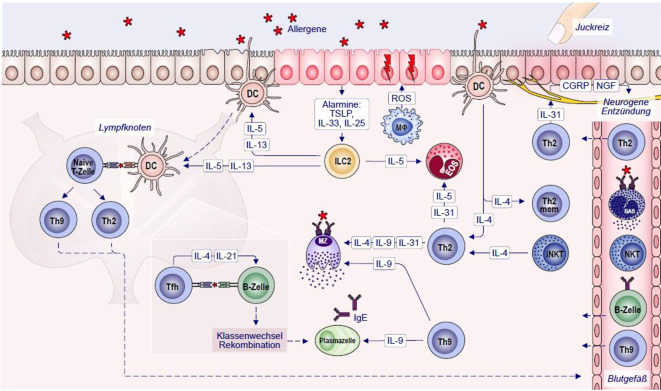
Mechanismen der Typ-IVb-Überempfindlichkeit (T2-Endotyp) bei der CRSwNP: Bei Überempfindlichkeitsreaktionen vom Typ IVb spielen Th2-Zellen eine zentrale Rolle, die durch Zytokine wie IL‑4, IL-13, IL‑5, IL‑9 und IL-31 angetrieben werden. Diese Zytokine stimulieren B‑Zellen zum Klassenwechsel zu IgE (IL‑4 und IL-13) und vermitteln Eosinophilie (IL-5), was zu Entzündungen und Gewebeschäden führt. Th9-Zellen, die sich durch IL‑4 und TGF‑β differenzieren, tragen erheblich zu dieser Reaktion bei, indem sie die IgE-Synthese erhöhen und das MC-Wachstum fördern. Die Reaktion wird durch ILC2-Zellen, MC und alternativ-aktivierte Mφ weiter erschwert. ILC2-Zellen, die durch IL-33 oder IL-25 aktiviert werden, arbeiten mit Th2-Zellen zusammen, produzieren Zytokine und beeinträchtigen die epithelialen Barrieren. Sie erleichtern die Rekrutierung von Eosinophilen und Basophilen und modulieren die APC-Funktion, was zur Chronifizierung von Typ-IVb-Reaktionen beiträgt. iNK-T-Zellen tragen zu dieser Reaktion bei, indem sie IL‑4 und IL-13 produzieren, die eine alternative Aktivierung in Mφ auslösen und die Entzündung fördern. Eosinophile wandern zu Entzündungsherden, aktivieren verschiedene Zytokine und Chemokine und setzen zytotoxische Granula frei, die zu Gewebeschäden, Nekrose und chronischer Entzündung beitragen. *AD* atopische Dermatitis; *AR* allergische Rhinitis; *B* B-Lymphozyt; *BAS* basophiler Granulozyt; *DC* dendritische Zelle; *EOS* eosinophiler Granulozyt; *EoE* eosinophile Ösophagitis; *sIgE* allergenspezifische Immunglobuline Klasse E; *ILC2* angeborene lymphatische Zellen vom Typ 2; *IL* Interleukin; *MC* Mastzellen; *Mφ* Makrophagen; *NKT* natürliche Killer-T-Zellen; *ROS* reaktive Sauerstoffspezies; *Th0/2/9* naive T‑Helfer-Lymphozyten/Typ 2 oder 9; *T2* Typ-2-Immunantwort

Typ-IVb-Reaktionen werden durch Th2-Zellen vermittelt, die ihren Phänotyp durch die Exposition gegenüber IL‑4, Basophilen oder NK‑T erwerben [42–45]. Th2-Zellen produzieren große Mengen an IL‑4, IL-13, IL‑5, IL‑9, IL-31 und Eotaxine I–III. IL‑4 und IL-13 sind die Schlüsselzytokine der Typ-IVb-Hypersensitivität und induzieren in B‑Zellen durch direkte (von IgM) und indirekte (von IgG 1) Mechanismen einen Klassenwechsel zu IgE [46]. IL-13 ist für den mit der Chronifizierung einhergehenden Gewebeumbau beim T2-Endotyp verantwortlich, während IL‑5 die Proliferation von Eosinophilen im Knochenmark, die zirkulierende Eosinophile und die Rekrutierung an den Entzündungsherden sowie ihr Überleben im Gewebe vermittelt, die durch Degranulation und Freisetzung von toxischen Eosinophilenprodukten chronische Gewebeschäden verursachen [47]. Th2-Immunantworten werden häufig von antigenspezifischen Th9-Zellen begleitet, die sich unter dem Einfluss von IL‑4 und Tumornekrosefaktor-beta (TGF-β) differenzieren [48]. Th9-Zellen können als wesentliche Akteure bei der Typ-IVb-Hypersensibilität angesehen werden. Sie produzieren IL‑9, das die IL-4-vermittelte Synthese von IgE durch B‑Zellen verstärkt und ein Wachstumsfaktor für Mastzellvorläufer im Knochenmark ist. Die Gedächtnisimmunreaktion bei Typ-IVb-Reaktionen wird unter anderem durch die Aktivierung von angeborenen Immunzellen wie Mastzellen, Basophilen, ILC2, Eosinophilen oder alternativ-aktivierten Mφ, verstärkt (Abb. 2; [49]). Zudem verstärkt TSLP die T2-Atemwegsentzündung [50]. ILC2-Zellen können Typ-2-Zytokine, insbesondere IL‑5, IL-13, IL‑9 und Amphiregulin, produzieren und einen Typ-2-Immunschutz gegen Helminthen vermitteln. ILC2 reagieren auf IL-33 und/oder IL-25 aus Epithelzellen. Sie stehen in Wechselwirkung mit Th2-Zellen, spielen eine Rolle bei der Rekrutierung von Eosinophilen und Basophilen und aktivieren APC, die die T2-Reaktion unterstützen [51]. ILC2 öffnen zusammen mit Th2-Zellen die epithelialen Barrieren durch IL-13 [52, 53].

Es wird vermutet, dass Mastzellen, Basophile und Typ-2-ILC die ursprüngliche Quelle von IL‑4 bei der Th2-Zelldifferenzierung darstellen. Darüber hinaus wird IL‑4 von einer einzigartigen Untergruppe invarianter natürlicher Killer-T-Zellen (iNK-T) (NK-T2) produziert, die über IL‑4 zur Aktivierung von CD4^+^- und CD8^+^-T2-Zellen sowie zur Auslösung und Aufrechterhaltung von T2-Entzündungen beitragen. Des Weiteren wurden in der nicht-IFN-γ-sezernierenden Gruppe IL-13-produzierende T2-NK- und NK-T-Zellen nachgewiesen, auch wenn es sich nur um eine kleine Fraktion handelt [54]. IL‑4 und IL-13 induzieren ein alternatives Aktivierungsprogramm in Mφ, das zu einem Suppressor für T1-verknüpfte zelluläre Aktivitäten wird. Eine Untergruppe von Mφ steuert T2-Funktionen an der Schnittstelle von Immunität und Gewebehomöostase und kann IL-13 produzieren [55].

Eosinophile sind die Hauptakteure bei allen Typ-IVb-T2-Immunreaktionen und tragen zu chronischen allergischen Entzündungen bei. Ausgereifte Eosinophile zirkulieren im Blut und wandern zu Entzündungsherden in allen entzündlichen Geweben des T2-Endotyps und zu Infektionsherden mit Helminthen. Eosinophile werden durch verschiedene Zytokine (wie IL-5) aktiviert, die von Immunzellen wie Th2 und MC freigesetzt werden. Sie werden auch durch Chemokine wie Eotaxin‑1 (CCL11), Eotaxin‑2 (CCL24), C‑C-Motiv-Chemokin-Ligand 5 (CCL5 oder RANTES), 5‑Hydroxy-Eicosatetraensäure und 5‑Oxo-Eicosatetraensäure sowie durch bestimmte Leukotriene wie Leukotrien B4 (LTB4) und Monozyten-Chemoattraktives Protein (MCP) 1/4 aktiviert [22]. IL-13 stimuliert den Austritt von Eosinophilen aus dem Knochenmark. Aktivierte Eosinophile setzen zytotoxische Granula frei, die Proteine wie das Major Basic Protein (MBP), das eosinophile kationische Protein (ECP), das Eosinophilen-Neurotoxin (EDN) und die eosinophile Peroxidase (EPO) enthalten. Diese Proteine können Gewebeschäden verursachen. Aktivierte Eosinophile produzieren zudem extrazelluläre DNA-Fallen, die aufgrund ihres Gehalts an eosinophilem Toxin Zellschäden verursachen ([56]; Abb. 2).

### Typ-IVc-Hypersensitivitätsreaktionen

#### T3-Immunantwort und T3-Endotyp

**Abb. 3:**
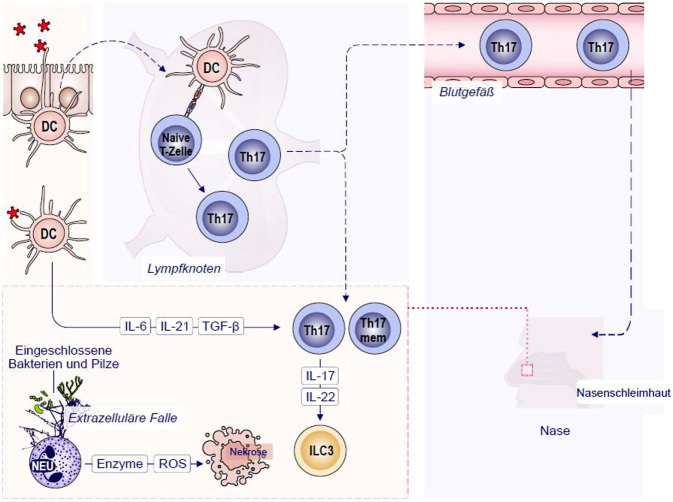
Mechanismen der Typ-IVc-Überempfindlichkeit in der Pathogenese der CRS: Bei der Typ-IVc-Hypersensitivität spielen Th17-Zellen und ILC3 eine Schlüsselrolle, indem sie Zytokine der IL-17-Familie produzieren, die die Rekrutierung von Neutrophilen fördern und die Produktion von Th2-Zytokinen steigern. Diese Reaktionen können zu Gewebeschäden durch Freisetzung von Enzymen und „neutrophil extracellular traps“ (NET) führen. *AD* atopische Dermatitis; *DC* dendritische Zelle; *IL* Interleukin; *ILC3* angeborene lymphoide Zelle vom Typ 3; *NET* neutrophile extrazelluläre Falle; *NK* natürliche Killerzelle; *ROI* reaktive Sauerstoffspezies; *TGF‑β* Transforming Growth Factor beta; *Th0/17* T-Helfer-Lymphozyten-naiver/17-Typ, *Th17 mem* Gedächtnis-Th17

Th17, Tc17, ILC3 und andere IL-17A- und IL-17F-produzierende Zellen sind an der neutrophilen Entzündung bei der CRS beteiligt. Beim Hypersensitivitäts-Typ IVc produzieren Th17-Zellen, die zur Helfer-T-Zelllinie gehören, Zytokine der IL-17-Familie. Diese regulieren angeborene Effektoren und orchestrieren lokale Entzündungen, indem sie die Freisetzung proinflammatorischer Zytokine und Chemokine induzieren, die sowohl Neutrophile rekrutieren als auch die Th2-Zytokinproduktion verstärken. Th17-Gedächtniszellen erwerben ihren Phänotyp durch die Exposition gegenüber IL‑6, IL-21, IL-23 und TGF‑β, die von APC bereitgestellt werden. Die wichtigsten von Th17-Zellen produzierten Effektorzytokine sind IL-17A, IL-17F, IL-21, IL-22 und GM-CSF [57]. IL-17A und IL-17F werden von CD4^+^- und CD8^+^-T-Zellen, γδ-T-Zellen und NK-Zellen als Reaktion auf IL-1β und IL-23 produziert. Ihre Standardfunktion ist, eine schützende Immunität gegen Pilze und Bakterien aufzubauen, indem sie die Produktion antimikrobieller Peptide, die Rekrutierung von Neutrophilen und eine verbesserte epitheliale Barrierefunktion fördern [58]. Neutrophile extrazelluläre Fallen (NET), Netzwerke extrazellulärer Fasern, die hauptsächlich aus DNA bestehen, schädigen zudem die sinunasale Mukosa (Abb. 3; [59]). IL-17A und IL-17F aktivieren ILC3 und Stromazellen zur Produktion von IL‑8, das Neutrophile an die Entzündungsherde lockt [60]. Die Infiltration des Gewebes durch neutrophile Granulozyten ist somit das Kennzeichen der T3-Immunantwort/des T3-Endotyps.

Der T3-Endotyp kann durch angeborene Immunzellen, insbesondere ILC3, verstärkt werden [61]. Entzündungen vom T3-Endotyp gehen häufig mit Reaktionen vom T1-Endotyp einher. Bei einigen Pathologien überwiegt jedoch die Aktivierung von Th17-Gedächtniszellen (Abb. 3).

### Andere mögliche Arten von Typ-IVb-Hypersensitivitätsreaktionen

Das p‑i-Konzept (pharmakologische Interaktion mit Immunrezeptoren) postuliert, dass einige Arzneimittel direkt und reversibel (nicht kovalent) an Immunrezeptoren binden können und dadurch die Zellen stimulieren. Ein bestimmtes Medikament kann an einen bestimmten T‑Zell-Rezeptor (TCR) oder direkt an ein bestimmtes HLA-Molekül binden, was die auffälligen HLA-Assoziationen bei einigen Überempfindlichkeitsreaktionen auf Medikamente erklären würde.

Th9-Zellen können in einigen Modellen die Immuntoleranz fördern [62] und vor parasitären Infektionen schützen [63], sie lösen aber auch allergische Entzündungen und Asthma aus [64], was ihre pleiotrope Rolle im Immunsystem unterstreicht. CD4^+^-T-Zell-Untergruppen (Th17, Th9), Mastzellen und ILC2 können IL‑9 produzieren. Neben anderen Effekten ist IL‑9 ein Schlüsselzytokin für die Th17- und Treg-Differenzierung [65], steigert die IL-4-vermittelte Produktion von IgE und IgG durch B‑Zellen [66] und fördert zusammen mit dem Stammzellfaktor das Wachstum von Mastzellen und Mastzellvorläufern im Knochenmark [67]. Th22 ist in der chronischen Phase am Gewebeumbau in den Atemwegen beteiligt [68–70]. Das prototypische Zytokin IL-22 zielt in erster Linie auf nichthämatopoetische Epithel- und Stromazellen ab, fördert deren Proliferation und spielt eine Rolle bei der Geweberegeneration. Darüber hinaus reguliert IL-22 die Abwehr an Barriereoberflächen.

## Diskussion und Schlussfolgerung

Die CRS weist in Europa und den USA eine Prävalenz von bis zu 11 % auf und gehört somit zu den häufigsten chronischen Erkrankungen überhaupt. Die Klassifizierung nach immunologischen Endotypen findet immer mehr Eingang in spezifische Krankheitsdefinitionen, wobei vor allem der T2-Endotyp häufig zitiert wird.

Die hier vorgestellte Nomenklatur ist für die Entwicklung des Fachgebiets von entscheidender Bedeutung. Der Ansatz basiert auf Krankheitsmechanismen und Endotypen statt auf Phänotypen und kann zur Entwicklung neuer Diagnoseinstrumente, verbesserter therapeutischer Strategien und einem besseren Krankheitsmanagement führen.

Unseres Erachtens liegt der Hauptvorteil dieser auf der Immunreaktion basierenden Nomenklatur in einem nuancierten Konzept, das Behandlungen in Richtung Präzisionsmedizin und personalisierte Medizin fördert. Das endgültige Ziel besteht darin, die Behandlung auf den einzelnen Patienten zuzuschneiden, und zwar auf der Grundlage spezifischer Immunreaktionen.

## Abkürzungen

APC: Antigenpräsentierende Zellen
CRS: Chronische Rhinosinusitis
CRSsNP: Chronische Rhinosinusitis ohne Nasenpolypen
CRSwNP: Chronische Rhinosinusitis mit Nasenpolypen
DC: Dendritische Zellen
EAACI: Europäische Akademie für Allergie und klinische Immunologie
ECP: Eosinophiles kationisches Protein
HLA: Humanes Leukozytenantigen
IFN: Interferon
Ig: Immunglobulin
IL: Interleukin
ROS: Reaktive Sauerstoffspezies
Th: T‑Helfer-Lymphozyten
TNF: Tumornekrosefaktor
TSLP: Thymisches stromales Lymphopoietin
